# Efficacy and predictability of maxillary and mandibular expansion with the Invisalign® system

**DOI:** 10.4317/jced.58315

**Published:** 2021-07-01

**Authors:** Maria-Luisa Vidal-Bernárdez, Ángel Vilches-Arenas, Boris Sonnemberg, Enrique Solano-Reina, Beatriz Solano-Mendoza

**Affiliations:** 1 Master’s Program in Orthodontics, University of Seville, Seville, Spain; 2 Department of Preventive Medicine and Public Health, Virgen Macarena University Hospital, University of Seville, Seville, Spain; 3 Private Clinic. Stuttgart, Germany; 4 Chairman, Department of Orthodontics, School of Dentistry, University of Seville, Seville, Spain; 5 Lecturer, Master’s Program in Orthodontics, University of Seville, Seville, Spain

## Abstract

**Background:**

The purpose of this study is to evaluate the efficacy and predictability of upper and lower orthodontic expansion with the Invisalign® system.

**Material and Methods:**

From a sample of 167 patients; 64 upper and 51 lower arches were randomly selected from patients who had been treated with plastic orthodontics (Invisalign® aligners, SmartTrack® material). Digital models were extracted at the beginning (ModT1) and at the end of treatment (ModT2) as well as the final ClinCheck® (CkT2). The canine, premolar and molar width was measured at the gingival and cuspid level of both arches, as well as the inclination of the upper first molar. Likewise, both arches were divided regarding the planned expansion at the level of the first molar into mild, moderate and severe.

**Results:**

The efficacy of expansion in the upper and lower arches showed a statistically significant difference (*p*<0.00005). During the measurements of predictability, around 98-100% was achieved at the coronal level and between 85-90% at the gingival level. Analyzing predictability regarding to the magnitude of expansion, superior and inferior moderate expansion, both gingival and cuspid, presented higher values.

**Conclusions:**

The Invisalign® system aligners (SmartTrack® material), proved to be a positive alternative for expansion movement offering high degree of predictability, both in the upper and lower arches. As a result, the most predictable level of expansion was moderate, having being the lower arch more foreseeable at the gingival level than the upper arch.

** Key words:**Predictability, Efficacy, Expansion, Aligner, Invisalign®.

## Introduction

Arch expansion is a treatment modality that solves transversal problems, where space is created in cases of crowding and changes in the shape of the dental arch are achieved. Principally, affecting smile aesthetics (1). Expansion is differentiated into orthopedic or dentoalveolar (2).

Dentoalveolar expansion is an option to treat transverse deficiency and/or crowding when they are mild. Improving the transverse dimension of the smile and correcting posterior crossbites whenever they are from dentoalveolar origin. However, when maxillary compression is moderate or severe and there is bone base involvement, orthopedic expansion techniques are applied (2,3). Dentoalveolar expansion can be carried out by means of fixed appliances or aligners, where the force is applied directly to the teeth and they undergo a displacement movement (4,5).

At the beginning of the 21st century, the first Invisalign® aligners (Align Technology, San Jose, CA, USA) appeared on the market, which demonstrated remarkable efficacy in dental movements, such as distalization, intrusion, extrusion and expansion, which were the source of studies by several authors (6-8). With regard to expansion, in its beginnings, it displayed a predictability of 40% and in later studies it increased to values close to 70% (9). Since 2013, the material of the EX30 aligners were replaced by a new SmartTrack® polymer (10).

The aim of our study is to evaluate the efficacy of upper and lower dentoalveolar expansion, as well as the predictability of the Invisalign system with the SmartTrack® material.

## Material and Methods

-Study design

A retrospective study was conducted, from which an initial sample of 167 patients who had been treated with the Invisalign® system, between March 2013 and December 2018, by a specialist was selected. The upper and lower STL models at the beginning of treatment (ModT1), at the end of treatment (ModT2) and the final ClinCheck® (CkT2) were analysed.

-Exclusion and inclusion criteria

We selected patients in the permanent dentition with erupted first molars who underwent expansion during Invisalign® treatment; models without attachments; patients with a minimum of 13 aligners, compliance with a full number of planned aligners and no changes in the middle; patients without the use of crossbite elastics and patients without dental agenesis (except for third molars).

On the other hand, we excluded patients with: a need of orthognathic surgery treatment, maxillary compression greater than 6 mm, a necessity of compression procedures during orthodontic treatment, all those models that had attachments, patients with lack of ModT1 and ModT2 model records, treatments of less than 13 aligners or that required the use of removable auxiliary appliances as well as the use of crossbite elastics and patients with dental agenesis.

-Sample size

Patients who met the previously defined inclusion/exclusion criteria were 64 in the upper arch and 51 in the lower arch, defining the definitive sample. The sample was divided into 3 groups regarding to the planned expansion related to the Table of movements at the level of the first molar, differentiated into mild (G1), moderate (G2) and severe (G3) (Table 1).

**Table 1:**
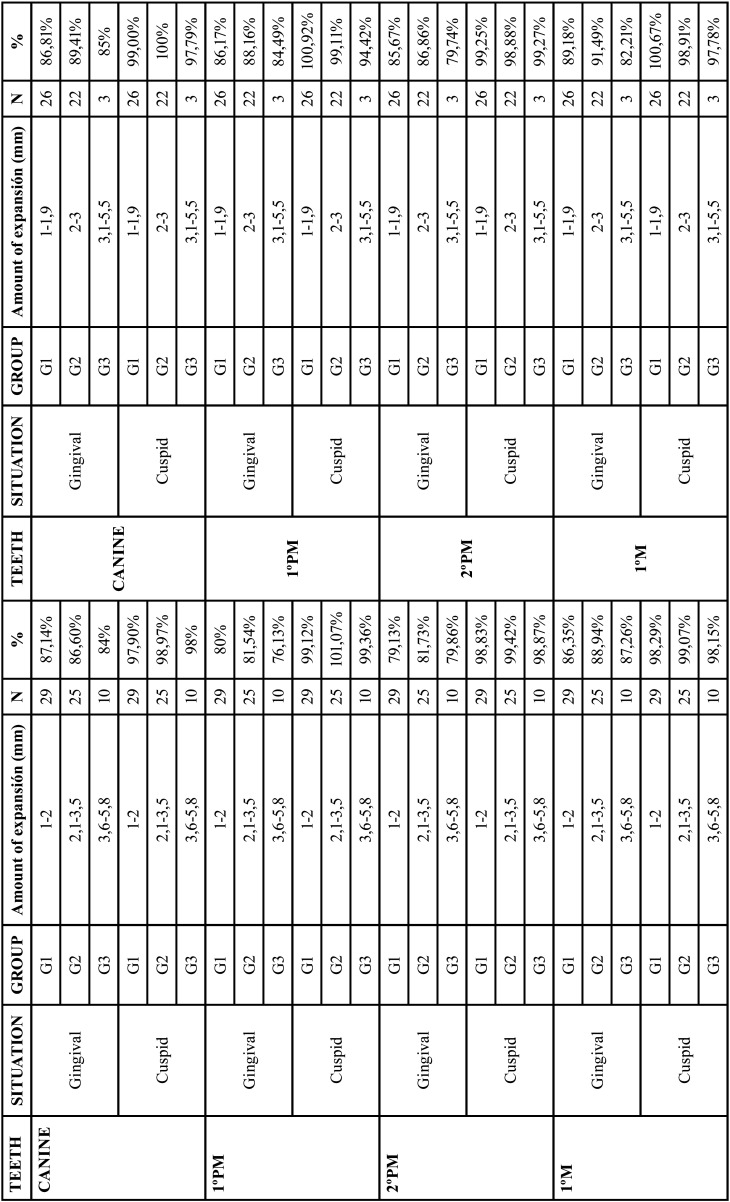
Predictability by groups according to the planned expansion of the upper 1st molars in the upper (left) and lower (right) arches.

Besides, the sample of the upper arch at the level of the first molar was divided according to the type of expansion planned in the Clincheck® movement Table, distinguishing between expansion by corono-vestibular torque (expansion at the level of the crown) and gresional movement (expansion at the level of the crown and root) (Table 2).

**Table 2:**
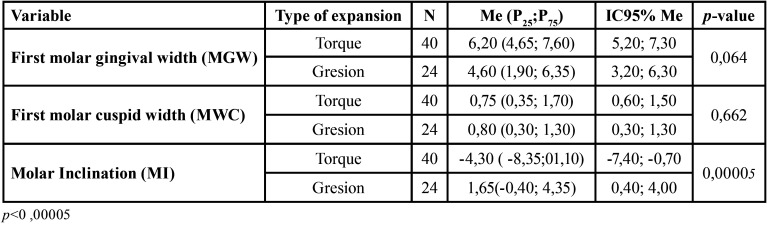
Comparison between post-treatment model measurements (Modt2) and post-treatment clincheck (CkT2) in the upper arch by groups according to the type of planned expansion in the 1st molars, group being torque expansion and group gresion expansion.

-Methodology

To measure the efficiency of the system, ModT1 and ModT2 were taken into account. And to measure the predictability ModT2 and CkT2, in case of patients with refinement, the first ClinCheck® of the refinement (instead of ModT2) was taken for the final situation, exporting it to STL for measurement. Both virtual models and ClinCheck® were analysed by using the analytical computer software NemosCast® (Nemotec, Madrid, Spain), which calibrates and performs measurements at real scale (1:1) on the three planes of space of the exported STL models. Reference points were identical for all upper and lower models.

**Figure 1:**
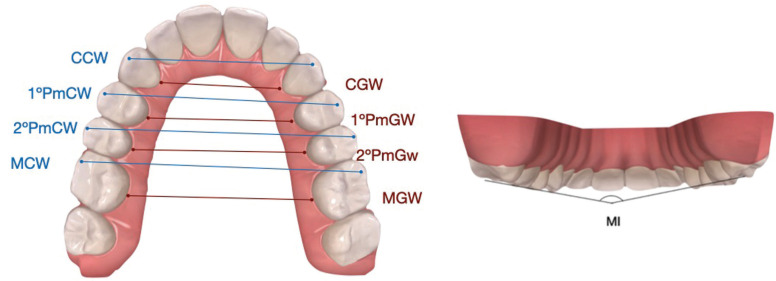
Linear measurements of gingival (G) and cuspid (C) widths of first and second premolar canines and first molars (Left). Angular measurement for the inclination of upper first molars (Right).

Gingival measurements: a hemiarch was taken from the center of the teeth’s gingival face (center of the palatal/lingual face in contact with the mucosa) to the same points of the contralateral hemiarch. The teeth selected were: canine (CGW), first and second premolar (1ºPmGW, 2ºPmGW) and molar (MGW) (Fig. 1).

Cuspid measurements: were taken from the cusp of the canine (CCW), vestibular cusp of the first and second premolar (1ºPmCW, 2ºPmCW) and mesiovestibular cusp of the first molar (MCW) of one hemiarch to the same points of the contralateral hemiarch (Fig. 1).

Molar inclination (MI): is the angle formed by the intersection of the lines passing through the distovestibular and mesiopalatal cusp of one molar and the contralateral one (Fig. 1).

-Statistical analysis

Numerical variables were expressed as mean and standard deviation values. To compare the means between two independent groups, Student’s t-test was performed for independent data once normality was validated (Shapiro Wilks test). Had the normality requirement not been met, the nonparametric test (Mann Whitney U-test) would have been applied for mixed quantitative-qualitative variables.

These analyses allowed the assessment of efficacy and predictability. Clinical predictability (%) was achieved by analyzing the medians for both gingival and cuspid widths, using the equation [(obtained/planned) *100].

To quantify the intraobserver measurement error, double measurements were performed on the pretreatment records of 30 randomized patients separated by a 2-week interval. All measurements were performed by a unique examiner in relation to the measurement of the models and ClinCheck®. To appraise intraobserver agreement, the intraclass correlation coefficient (ICC) was used.

All statistical comparisons were fixed at the level of statistical significance of *p-value* < 0.00005 and a 95% confidence interval. The data were analyzed by an intention-to-treat approach, using SPSS 26.0 software for Windows (SPSS Inc.Chicago, IL, USA).

## Results

-Calibration 

The results obtained displayed a high degree of intraobserver reproducibility with a t-test *p* > 0.00005 and intraclass ratio coefficient of ICC > 0.80.

-Efficacy. Pre-treatment digital model versus Post-treatment digital model (ModT2-MoDT1) 

In the upper arch, the greatest change was at the gingival level of premolars (with a mean of 3.36 and 3.42 mm in first and second molars, respectively) and the least change was at the cuspid level of these same teeth, with a difference of 1.53 and 1.60 mm, accordingly. All the data studied showed a *p-value* <0.00005, and all the changes produced were statistically significant.

Contrary, in the lower arch, the greatest change was at the gingival level of the 2nd premolar, 3.44 mm and the least at the cuspid level of the 1st premolar, 1.24 mm. As in the upper arch, all the data showed a value of *p* < 0.00005.

In absolute terms, the changes produced in the upper arch are greater than those produced in the lower arch (Table 3).

**Table 3:**
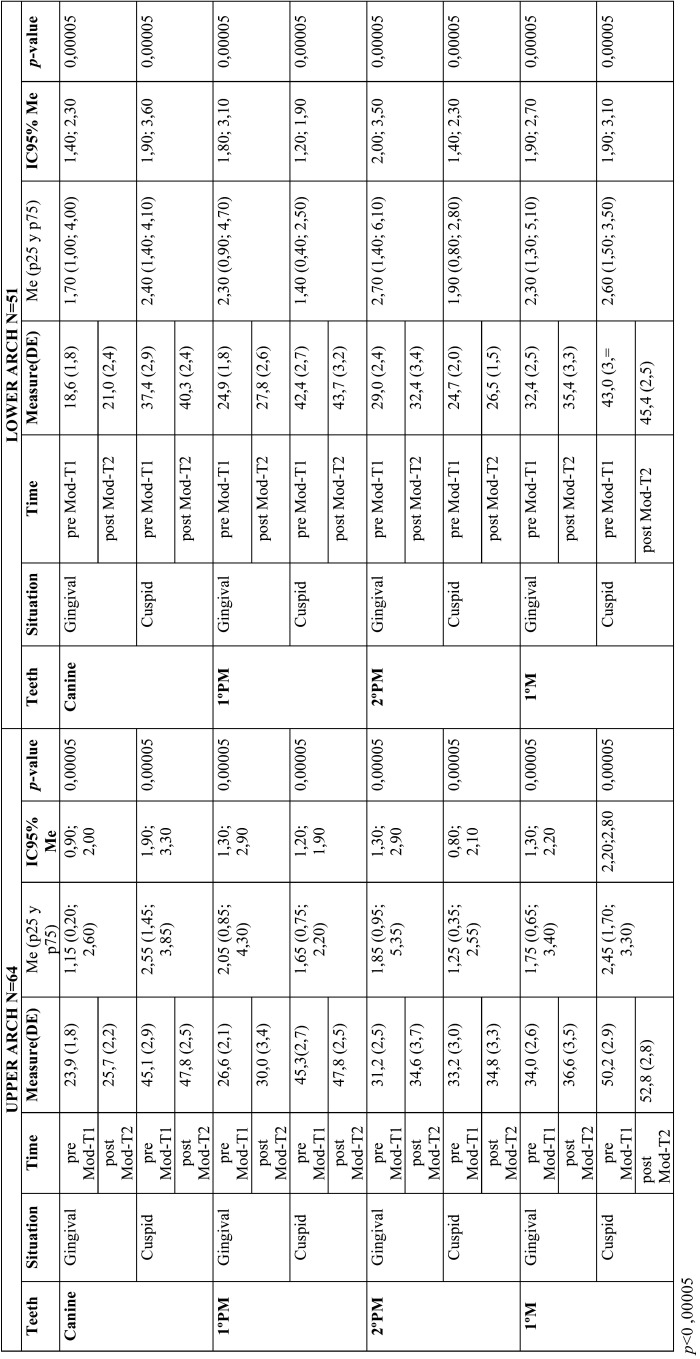
Efficacy. Pre-treatment digital model versus Post-treatment digital model (ModT2-MoDT1) in the upper (left) and lower (right) arch.

-Predictability. Post-treatment digital model versus final ClinCheck® (ModT2-CkT2) 

For the upper arch values, with the exception of the width of the 1ºpm cuspid (*p*=0.304), the data studied showed a *p-value* < 0.00005 and all the changes produced were statistically significant. At the gingival and cuspid level, the predictability for canines was 87.71% and 98.35% respectively. Obtaining for first premolars a result of 84.03% and 99.36%, for second premolars 84.28% and 100.58% and for first molars 87.35% and 98.32% (Table 3).

Whereas, in the lower arch, with the exception of the cuspid width at the level of the teeth measured, the data studied displayed a *p-value* < 0.00005, with the changes in gingival widths being the only statistically significant. Analyzing the predictability percentages, 88.98% at the gingival level and 99.26% at the cuspid level were obtained in the canines, 88.82% and 100% in the first premolars, 90.50% and 100.38% in the second premolars, and 90.31% and 99.78% in the first molars, respectively (Table 4).

**Table 4:**
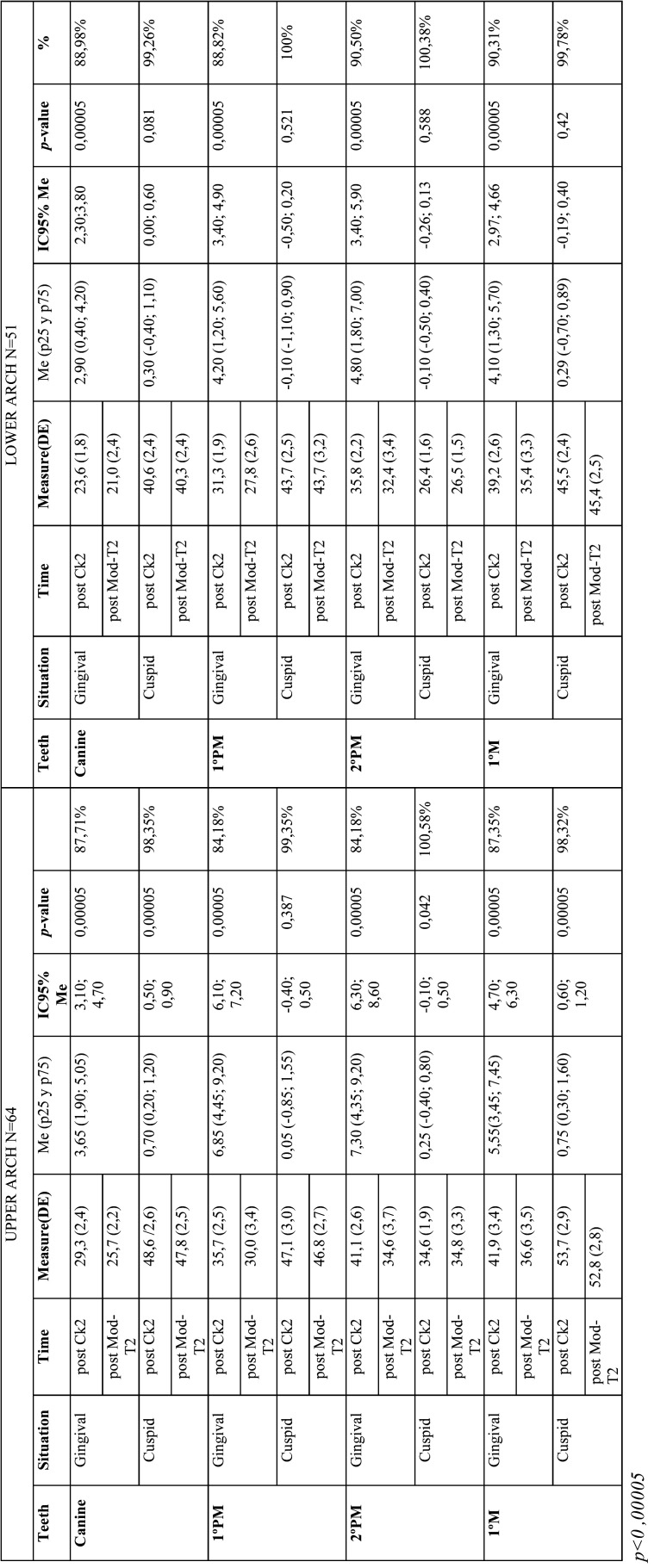
Predictability. Post-treatment digital model versus final ClinCheck® (ModT2-CkT2) in the upper arch (left) and lower arch (right).

Analyzing the predictability by groups, upper and lower, both gingival and cuspid, G2 presented higher values than the other groups (Table 1).

-Torque Corono-Vestibular vs Gresional Expansion.

The difference of the first molar widths both gingival and cuspid presented values of *p*=0.064 and *p*=0.662, respectively. Assuring that there is no statistically significant change between them. On the contrary, the molar inclination produced between both groups is statistically (*p*=0.00005) (Table 2).

## Discussion

Through the present study, cases treated by the same professional with experience in the technique were compared, thus eliminating interprofessional bias variables. A unique operator performed all the measurements, having been also statistically validated in the work he performed; By doing so, we assumed the measurements are accurate and comparable with each other (ICC>0.80). The measurements studied were of the dental type; however, in order to know the real changes in the transversal dimension, skeletal measurements should have been made with 3D tomographic images (CBCT). These were ruled out for questionable ethical reasons due to the radiation dose received by the patients.

The new SmartTrack® material (LD30) provides several advantages compared to the old material (Ex30), such as higher soft and constant force, higher elasticity, chemical stability and a more precise and comforTable aligner fit. (11,12)

In 2009, Kravitz *et al*., was the first group to study the efficacy of tooth movement with the Invisalign® system, where they observed that the average accuracy of tooth movement was 41% and, at the canine level, more specifically 36% in the upper arch and 29.9% in the lower arch, collecting predictability results much lower than those presented in this study (8).

In 2017, Solano-Mendoza *et al*. obtained in their study that the initial ClinCheck® is an exact reproduction of the pretreatment model, therefore showing the efficacy of the impression. And in this study, the comparison between CkT1 and ModT1 was not performed, using exclusively initial position ModT1. The average expansion obtained in this study at the canine level was 0.54 mm, 1stPm 1.39 mm, 2ndPm 1.25 mm and 1st molar 0.56 mm (measuring from gingival which is the closest to the true mass expansion) (13).

As shown in the results, at gingival level 1.72 mm was achieved in canines; in 1st premolar 3.36 mm; 2nd premolar 3.42 mm and in 1st molar 2.66 mm (Table 3). Observing slightly higher values than in the previous study, and being able to appreciate an increase in the efficacy with the new material.

To determine the efficacy of expansion with Invisalign®, a comparison was made between ModT1 and ModT2, generally achieving more cuspid expansion than gingival expansion, typical of the expansion due to torque correction of the upper arch (14). (Table 3). Nevertheless, in the lower arch, the premolars presented more changes at the gingival level than at the cuspid level, this could be due to the meshing of the upper arch with the lower arch interfering with the cuspid expansion.

In 2017, Houle *et al*. obtained in their study that the prediction at the gingival and cuspid level in canines was 67.8% and 88.7%, for first premolars 67.7% and 84.7% for second premolars 62.3% and 81.7% for first molars 52.9% and 76.6% respectively (15). The Invisalign® system becomes less accurate as we move from the anterior to the posterior region (8,13,15) and likewise in our study the first molar was the least predictable tooth.

**Figure 2:**
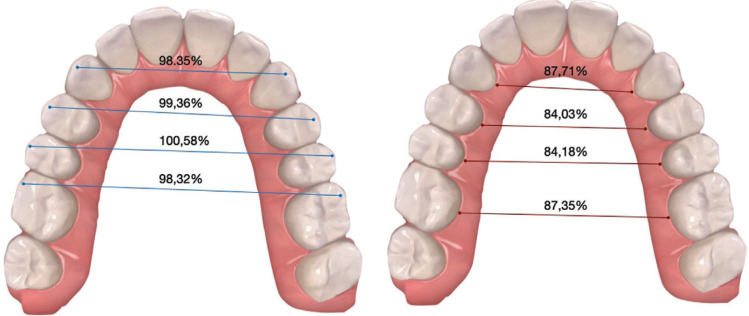
Predictability measurements of upper arch expansion at the cuspid (Left) and gingival (Right) levels.

With regard to the above statements, the predictability achieved in this study for the upper arch at the gingival and cuspid level for the canines was 87.71% and 98.35%, for the first premolars 84.03% and 99.36%, for the second premolars 84.28% and 100.58%, and for the first molars 87.35% and 98.32% respectively. The predictability obtained at the gingival level was lower than at the cuspid level, this phenomenon results from the influence of the aligners on the crowns, being thus the movements more predicTable in patients who present wide and large crowns (6) (Fig. 2). This have been observed in all groups regardless of the amount of expansion. Analyzing the predictability by groups, it could be said that G2 (moderate expansion) is more predicTable than G1 and G3, with no statistically significant differences between them (Table 1).

In 2020, Morales *et al*. studied the efficacy and predictability of expansion with the Invisalign® system in the upper arch, being the only study, along with the one performed, carried out with the SmarTrack material. These authors presented a sample of 114 patients and compared the width of canines, premolars and molars at the cuspid level (16). The efficacy was for canines 1.87mm, 1st premolars 3.14mm, 2nd premolars 3.45mm, 1st molars 2.57mm and 2nd molars 0.45mm, obtaining similar results to our study but with higher values. It may be due to the fact that their sample was based on the presence of teeth with more negative torques. The predictability for canines 79.1%, 1st premolar 79.9%, 2nd premolar 80.9%, 1st molar 79.9% and 2nd molar 71.9%. The lowest efficacy and predictability is at the level of the second molar, which was not measured in our study, but similar results were obtained for the rest of the teeth evaluated. Unlike our study, the measurements were performed on grids (calibrated to 1 mm) in 2D images, hence being less accurate than performing it on 3D STL models (at 1:1 scale). Similarly, expansion was not evaluated at the gingival level, only at the coronal level, meaning that expansion was only considered by a tilting movement of the crown.

**Figure 3:**
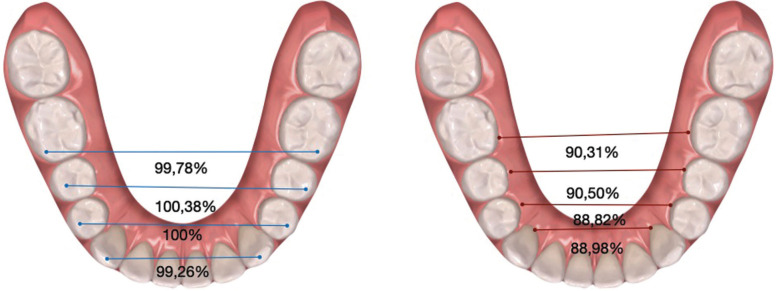
Predictability measurements of lower arch expansion at the cuspid (Left) and gingival (Right) levels.

In 2018, Charalampankis *et al*. found no statistically significant differences between the horizontal movements predicted by Clincheck® with the movements achieved, but presented a predictability of the transverse dimension, being higher in the lower arch (95-97 %) than in the upper arch (77-78 %) (15,17). Similarly, in our study the overall lower arch predictability was 88%, 98.9% at the cuspid level and 76.4% at the gingival level. This better result can be explained by the fact that the amount of change requested in the lower arch is usually less than in the upper arch. In addition, the resistance is reduced since the upper arch is expanding simultaneously, the same is reflected in the results, although with a higher predictability in general. Showing the improvement of the system with the new material. Our results present a predictability for the lower arch in canines at the gingival and cuspid level of 88.98% and 99.26% respectively, in first premolars of 88.82% and 100%, in second premolars of 90.50% and 100.38% and in first molars of 90.31% and 99.78% (Fig. 3, Table 4) In the lower arch, predictability at the gingival level is lower than at the cuspid level, as in the upper arch.

Both the study group of Charalampakis *et al*. and Papadimitriou *et al*. report that the use of SmartForces® features could be more effective for certain movements, including posterior tooth expansion (17,18). As Kravitz *et al*., Houle *et al*. observed that the prediction of expansion by orthodontics with aligners has an implication for teeth inclination in addition to vestibulolingual translational movement (8,9).

Zhoua N *et al*. and Grünheid *et al*. corroborated the above conclusions, arguing that aligners could increase interarch width, but expansion was mainly achieved by tilting movement. Zhoua N *et al*. carried out the first study in which the changes in expansion with the Invisalign® system were evaluated using 3D tomographic images (CBCT) in which results obtained were that the expansion efficiencies at the coronal level were 79.75% at the canine level, at the first and second premolar level 76.10% and 73.27% respectively and at the first molar level 68.31% (19,20).

On the other hand, Zhoua N *et al*. also documented that the efficiency of the expansion movement in gresion for the maxillary first molar was 36.35% (19). Should be noted that the aligner, being flexible, may camouflage some buccal inclination, because a ClinCheck® reflects a body movement that occurs partially. To resolve this ambiguity, in our study, we compared the gingival and cuspid width of the upper first molar planning torque and gressional expansion (Table 2). We concluded that it is just as effective to plan for gresional expansion as for torque, essentially there is no statistically significant difference. Notwithstanding, there is a significant change in the inclination of the molars, with a difference of 6 degrees. Therefore, an additional change in inclination should be incorporated in the aligner to achieve a more parallel dental movement.

The limitations of the study include the non-inclusion of the 2nd molars because not whole of the sample analysed had it. Nonetheless, since it is a terminal tooth, it would be interesting for future research to include it as well as to study the expansion obtained with the use of crossbite elastics and horizontal attachments beveled to occlusal (HBO).

Moreover, there would be the chance of expansion studies with CBCT to observe the tipping of the molars pre and post expansion, despite the fact it is ethically questionable to use some unnecessary radiation to perform such a measurement.

## Conclusions

1. The efficacy of expansion at the level of cuspid and gingival widths (canines to molars) in both the upper and lower arches is statistically significant (*p*< 0.00005).

2. It is equally effective to plan for gresional expansion as by torque. There is no statistically significant difference in the efficacy of both.

3. The data reflecting the predictability of the expansion of the Invisalign® system with the SmarTrack® material showed a high degree of predictability (coronal 98-100% and gingival between 85-90%).

4. The most predicTable expansion is the moderate expansion. In our study, it is the one within group 2 (expansion between 2-3.5 mm) in both arches and comparing them between both arches.

5. Expansion in the lower arch is more predicTable at the gingival level than in the upper arch.

6. Expansion is more foreseeable at the coronal level than at the gingival level. Hence expansion achieved only with the aligner is a coronal movement.
